# 

**Published:** 1911-02-25

**Authors:** 


Telegraphic Address: Ospedale, London.

THE MODERN MEDICAL AND INSTITUTIONAL JOURNAL.

Telephone Number: 2734 Gerrard.

NEW SERIES. No. 212, Vol. VIII.

No. 1284, Vol. XLIX.

SATURDAY, FEB. 25TH, 1911.

PRICE THREEPENCE

